# Effect of texturised soy protein and yeast on the instrumental and sensory quality of hybrid beef meatballs

**DOI:** 10.1007/s13197-018-3552-9

**Published:** 2019-02-25

**Authors:** Simona Grasso, Gabrielle Smith, Sophie Bowers, Oluseyi Moses Ajayi, Mark Swainson

**Affiliations:** National Centre for Food Manufacturing, College of Sciences, University of Lincoln, Lincoln, UK

**Keywords:** CATA, Consumer sensory analysis, Hybrid meat product, Texturised soy protein, Yeast, Beef meatballs

## Abstract

The study aimed to investigate the effect of introducing texturized soy protein (TSP) at different levels (15% and 30%) with and without nutritional yeast as flavour enhancer on the sensory and instrumental quality of beef meatballs, compared to a soy and yeast-free control. Proximate analysis, colour, instrumental texture, cook loss, and sensory quality were investigated. Sixty participants assessed the samples using Check-all-that-apply (CATA) questions and hedonic scales. Overall, the texture of all TSP-containing samples received significantly higher acceptability scores than control, while 15% TSP with yeast received the highest flavour and overall acceptability scores. Penalty-lift analysis of CATA terms identified the main drivers for liking as “moist looking”, “juicy”, “soft” and “crumbly and easy to cut”. Control samples were significantly more often associated than the other recipes to the term “hard”, a key driver for dislike and the least associated to “soft” and “crumbly and easy to cut”. Adding 15–30% TSP with or without yeast inclusion could be beneficial for the development of future meat hybrids with acceptable sensory quality.

## Introduction

The consumption of red and processed meat has recently been associated to cancer, with red meat classified as “probably carcinogenic” and processed meat as “carcinogenic” (International Agency for Research on Cancer 2015). These conclusions reached the scientific community, but also the general public through mass media (Domingo and Nadal 2017). Media coverage of the potentially negative side effects of meat consumption seem to play a major role in reducing consumer meat intake (Cordts et al. 2014). A recent Dutch survey found that 77% of consumers considered themselves to be meat-reducers and not avoiders (Dagevos and Voordouw 2013). A recent market research study in the UK (Mintel 2017), reported that over a third (35%) of meat and poultry eaters and buyers have regular days when they avoid meat, rising to 43% among consumers who are 25–34 years old. In January 2018, the retailer Waitrose in the UK launched a range of sausages, meatballs and burgers containing up to 35% fruit, vegetables or pulses, specifically targeting consumers looking to reduce their meat intake as part of their healthier food launches (Waitrose 2018).

Flexitarianism, defined by Raphaely and Marinova (2014) as “part-time vegetarianism” or as “the reduction in individual meat consumption to the recommended healthy dietary guidelines”, could open new market opportunities for the meat industry. As Hicks et al. (2018) suggest, “it would be efficient and wise for the meat industry to build a strategy around the flexitarian demographic, to ensure their needs are met and to keep them consuming meat, rather than risk losing them to veganism”.

Hybrid meat analogues, meat products whereby a proportion of meat has been partially replaced by more sustainable protein sources, could bridge the gap between meat and meat-free products, providing convenience, and allowing consumers to continue using meat products as they conventionally would (Neville et al. 2017). Hybrid sausages, hamburgers, and mince have already entered the Dutch food markets and have created a means whereby eating hybrid meat products gradually becomes more accessible (de Bakker and Dagevos 2012).

Within this context, the aim of this work was to investigate the effects of substituting 15% or 30% beef mince with texturised soy protein (TSP) in beef meatballs, with and without nutritional yeast addition. Nutritional yeast consists of whole yeast cells from *Saccharomyces cerevisiae* which have been inactivated by heat and then roller drum dried to obtain powders or flakes (Methven 2012). Nutritional yeast was used in the current study as a flavour enhancer and source of umami taste compounds (Dermiki et al. 2013), as the addition of TSP alone has been reported to lower the flavour score, reduce the beef flavour and the overall flavour quality in beef burgers (Angor and Al-Abdullah 2010). TSP is already a popular non-meat ingredient used by the meat industry (Feiner 2006), for its functional properties such as water-binding and fat-binding ability, enhancement of emulsion stability and increased yields, with relatively low cost compared to lean meat (Chin et al. 2000). However, the effects of its addition have been contradictory on occasions, due to the variable nature of the material, processing conditions and meat matrix used (Colmenero 1996). In general, high levels of soy inclusion are associated with an increase in tenderness and juiciness, with improvements in texture and moisture retention, but also with decrease in cook loss, calorific value and meat aroma (Feiner 2006; Keeton 1994). TSP has been used for meat replacement in previous studies (Deliza et al. 2002; Kassama et al. 2003; Kilic et al. 2010), however there are no reports of use of TSP in combination with yeast as a flavour enhancer. Therefore, the impact of such reformulation on the instrumental and sensory quality of beef meatballs was investigated.

## Materials and methods

### Meatball preparation

Lean ground beef (4.5 ± 0.1% fat), texturized soy protein (TSP, Neal’s yard wholefoods Ltd., UK), yeast flakes (dried inactive yeast, Engevita^®^, Lallemand, UK), bread crumbs (natural breadcrumbs, Tesco, UK) and salt were purchased from local shops. Formulations used for the preparation of beef meatballs are shown in Table 1. Five treatments of meatballs were prepared: control (100% beef), TSP15 and 30 (15% or 30% of beef replaced with hydrated TSP granules, respectively), TSPY15 and 30 (15% or 30% of beef replaced with TSP granules hydrated with water and yeast, respectively). Substitution levels of 15% and 30% were used based on the work from Deliza et al. (2002), who used 15% and 30% TSP to produce ground beef patties with and without colorants. All treatments contained the same amount of breadcrumbs (used as a binder) and salt. TSP granules were hydrated in hot water (85 °C) in a ratio of TSP: water of 1:1.5 (w/w) for 5 min. In the yeast containing samples, yeast flakes were added at 10% of the combined TSP and water weight. This level of yeast inclusion was determined by preliminary trials as the optimal amount of yeast to be used. Ground beef and other ingredients were mixed in a food processor (Kitchen Aid Artisan, model 5K5M150) for 1 min. The mix was then shaped by hand into 3 cm diameter round shaped meatballs weighing approximately 15 g each. Meatballs were then vacuum packed and frozen at − 20 °C until required for analysis.

**Table 1:** Formulations (%) used to manufacture the five meatball treatments

Ingredients	Control	TSP15	TSPY15	TSP30	TSPY30
Beef	94.25	79.25	79.25	64.25	64.25
Hydrated TSP	0	15	0	30	0
Hydrated TSP with yeast	0	0	15	0	30
Bread crumbs	5	5	5	5	5
Salt	0.75	0.75	0.75	0.75	0.75

C: 100% beef, TSP15: 15% soy substitution, TSPY15: 15% soy substitution + yeast, TSP30: 30% soy substitution, TSPY30: 30% soy substitution + yeast

The meatballs were cooked from frozen using a preheated commercial kitchen oven (SMEG, model SUK62CMX5) at 200 °C for 25 min, until an internal temperature of 75 °C was achieved. The temperature was monitored with a digital thermometer (Hanna, model HI9241). The physicochemical measurements (proximate, yield, colour and texture) were carried out in triplicate, on three batches of meatballs (1.5 kg per batch) manufactured on three different days. Samples were left to equilibrate at room temperature for about one hour before the physicochemical measurements were carried out.

### Proximate analysis

Moisture, protein, fat and ash were quantified according to ISO standards 1442:1997 (ISO 1997), 937:1978 (ISO 1978), 1444:1996 (ISO 1996) and 936:1998 (ISO 1998), respectively.

### Cooking yield measurement

Cooking yield was determined by measuring the weight of fifteen meatballs for each treatment and for each replicate. The difference in weight, at room temperature, before cooking (and before freezing) and after cooking, was calculated using the below equation from Gök et al. (2011):

$$ Cooking \,yield\, \left( \% \right) = \frac{cooked \,weight}{raw \,weight} \times 100 $$

### Colour

The colour of meatballs was measured using the Hunter Lab system (L*: lightness; a*: redness/greenness; and b*: yellowness/blueness) with a colorimeter (Konica Minolta CR-400), calibrated with a white tile (Minolta calibration plate, No. CR-A43), at 2° observation angle with a C illuminant source (Y = 93.5, x = 0.3114, y = 0.3190). Five cooked meatballs were used per treatment and per replicate. After cooking, samples were allowed to equilibrate to room temperature. Then each meatball was sliced in half and two internal colour readings per side were taken by placing the lens of the colorimeter in contact with each meatball section. Measurements were automatically captured using the Colour Data Software (CM-S100w, SpectraMagic NX, Konica Minolta). The overall difference in colour was calculated using the below formula (Francis and Clydesdale 1975):

$$ \Delta E = \left[ {\left( {L^{*} - L_{0}^{*} } \right)^{2} + \left( {a^{*} - a_{0}^{*} } \right)^{2} + \left( {b^{*} - b_{0}^{*} } \right)^{2} } \right]^{0.5} $$

### Texture profile analysis

Texture profile analysis (TPA) was performed according to the procedure of Bourne (1978) using a Texture Analyser (TA-XT Plus model, Stable Micro Systems, Godalming, Surrey, UK) with a trigger force of 5 g. The TPA tests were carried out using a cylindrical probe (SMS P/75; 75 mm diameter compression platen). The pre-test and test speeds of 1 mm/s and 5 mm/s were used for the TPA, respectively. Sample hardness, springiness, cohesiveness and chewiness were measured automatically using the Exponent Software (version 6.1.9.0) as the cylindrical probe compressed each sample to a depth of 15 mm in a two-loading cycle. The 15 mm compression depth ensured that the degree of compression for the test was at least 50%, in order to mimic the large deformation in the mouth. Cooked whole samples were used and tests were performed at ambient temperature on ten samples per recipe and per replicate.

### Sensory evaluation

Ethical approval for this study was granted by the College of Science Research Ethics Committee of the University of Lincoln (approval number UID CoSREC496). The sensory evaluation was carried out in a sensory laboratory designed according to ISO 8589 (ISO 2007). Samples were cooked from frozen at 200 °C for 25 min, as this cooking time and temperature allowed a temperature of 75 °C for at least 2 min to be reached in the centre of the samples. The samples were wrapped in aluminium foil and placed in a lidded Pyrex dish in the oven at 70 °C no longer than 15 min to keep warm until their evaluation. Samples were served on white paper plates coded with 3-digit random numbers and in randomised balanced design. Water was used as a palate cleanser between samples.

Sixty panellists were recruited from the University of Lincoln, Holbeach campus, UK, based upon being regular consumers of beef meatballs. Panellists were presented with a total of five samples in a sequential monadic order (Kemp et al. 2011) and all samples were evaluated in a single testing session. Panellists were asked to first cut the sample in half and assess its appearance using a 9-point anchored scale going from “extremely like” to “extremely dislike” (Peryam and Pilgrim 1957). Then panellists were presented with check-all-that-apply (CATA) terms relating to appearance and were asked to select all the terms that they considered appropriate to describe the sample. The panellists were then asked to taste the sample and assess the flavour and texture using the 1–9 liking scales and the related CATA terms. Finally panellists were asked to rate the overall quality of the sample on a 1–9 liking scale. The twenty-four CATA terms used were divided into three categories: appearance (“moist looking”, “dry looking”, “uniform colour (outside)”, “uneven colour (outside)”, “dark brown (inside)”, “light brown (inside)”, “unusual”, “characteristic”), texture (“juicy”, “dry”, “hard”, “soft”, “solid and difficult to cut”, “crumbly and easy to cut”, “unusual”, “characteristic”), and flavour (“tasty”, “bland”, “cheesy”, “weak meaty”, “strong meaty”, “wheat-cereal like”, “unusual”, “characteristic”). The terms used in CATA questionnaire were chosen from the literature available on meat products (da Conceição et al. 2015; Grasso et al. 2017; Neville et al. 2017). CATA terms were randomised using a “to assessors” allocation (Meyners and Castura 2014), therefore the order of CATA terms was stable across samples for each assessor, but it was randomised across each of the sixty assessors.

### Statistical analysis

Analysis of variance (ANOVA) was performed to analyse the physicochemical data, with treatments as fixed effect and the experiment replications as a random term (*n* = 3). When significant differences were found (*P* < 0.05), the means were separated using Tukey’s test. Contingency tables were generated for the CATA data by counting the frequency of use of each term for each sample and Cochran’s Q test with post hoc analysis was conducted via multiple pairwise comparisons. Correspondence analysis (CA), was performed on the CATA data to visualise the five samples and CATA terms using χ^2^-distances. Penalty-lift analysis was carried out calculating the difference between the average liking across assessors when one CATA term was selected minus the average liking across assessors when the same CATA terms was not selected (Meyners and Castura 2014). Statistical analyses were performed using Excel 2016 (Microsoft Co.) and SPSS (version 24) statistical software (IBM Inc. Chicago, IL, USA). Randomisation of CATA terms and sample serving order were carried out using RedJade sensory software (Boulder, Colorado, USA).

## Results and discussion

### Proximate analysis

The results of the proximate analyses of the five treatments are shown in Table 2. There were no significant differences in the protein and ash contents across the five recipes. The addition of the TSP, containing 50% of protein, nutritionally balanced the overall protein content, despite the beef protein removal. This is in accordance with a study by Kilic et al. (2010) on beef kofte, where no effects in the protein content were reported with up to 20% soy inclusion. Soy addition had an effect on fat, with the total fat content tending to go down with increasing soy substitution. A 30% substitution resulted in a significant 25–27.5% decrease in fat content compared to control, while a 15% substitution resulted in 6–12% decrease in fat compared to control (non-significant). This was expected, as the TSP used contained 1% fat, while the lean beef used contained about 4.5% fat. Differently from our results, Kilic et al. (2010) found no significant differences in fat content with up to 20% soy substitution. Although in their study lean beef mince was also used (4 ± 0.6%), it is possible that their TSP contained higher levels of fat which might have balanced the removal of beef fat. Moisture was also effected by the soy addition, with TSP15, TSPY15 and TSPY30 resulting in significantly lower moisture values than control. TSP30 had lower moisture content than control (but not significantly lower), probably because of the large standard deviation of control samples.

**Table 2:** Proximate analyses, yield, colour and texture across the five recipes

Parameter	Control	TSP15	TSPY15	TSP30	TSPY30
Proximate					
Moisture (%)	64.28 ± 0.84^a^	60.96 ± 0.93^b^	60.95 ± 0.49^b^	61.34 ± 0.41^ab^	59.48 ± 0.17^b^
Protein (%)	24.09 ± 0.20^a^	24.82 ± 0.49^a^	25.19 ± 0.65^a^	23.42 ± 0.45^a^	23.94 ± 0.26^a^
Fat (%)	4.29 ± 0.25^a^	4.02 ± 0.33^ab^	3.77 ± 0.10^ab^	3.11 ± 0.09^b^	3.21 ± 0.12^b^
Ash (%)	1.82 ± 0.08^a^	2.01 ± 0.10^a^	1.92 ± 0.32^a^	2.30 ± 0.17^a^	2.30 ± 0.10^a^
Yield (%)	83.74 ± 0.38^b^	83.54 ± 0.24^b^	84.91 ± 0.33^b^	88.43 ± 0.32^a^	88.52 ± 0.26^a^
Internal colour					
L*	47.9 ± 0.3^a^	47.7 ± 0.4^a^	48.2 ± 0.4^a^	46.9 ± 0.3^a^	47.3 ± 0.2^a^
a*	10.3 ± 0.2^a^	9.4 ± 0.1^b^	9.7 ± 0.1^b^	9.4 ± 0.1^b^	9.3 ± 0.1^b^
b*	10.1 ± 0.2^b^	10.7 ± 0.3^b^	12.1 ± 0.2^a^	12.1 ± 0.2^a^	12.8 ± 0.3^a^
ΔE	–	1.1	2.4	2.1	2.9
External colour					
L*	40.5 ± 0.3^b^	41.0 ± 0.3^ab^	40.9 ± 0.4^ab^	41.6 ± 0.4^ab^	42.1 ± 0.3^a^
a*	10.9 ± 0.1^a^	9.4 ± 0.1^bc^	9.9 ± 0.1^b^	8.9 ± 0.2 ^cd^	8.7 ± 0.2^d^
b*	11.1 ± 0.1^c^	12.3 ± 0.2^b^	12.7 ± 0.1^b^	12.9 ± 0.2^b^	13.6 ± 0.2^a^
ΔE	–	2.0	2.9	2.0	3.8
Texture					
Hardness (N)	11,244.02 ± 476.62^a^	11,101.09 ± 492.93^a^	9789.17 ± 593.22^ab^	8488.95 ± 354.46^b^	8985.59 ± 395.91^b^
Springiness (mm)	0.78 ± 0.01^a^	0.75 ± 0.01^b^	0.74 ± 0.01^b^	0.76 ± 0.01^ab^	0.74 ± 0.01^b^
Cohesiveness	0.44 ± 0.01^a^	0.42 ± 0.01^b^	0.42 ± 0.01^b^	0.44 ± 0.01^a^	0.42 ± 0.01^ab^
Chewiness (mJ)	3814.22 ± 140.70^a^	3484.19 ± 152.06^ab^	3007.20 ± 177.56^bc^	2823.55 ± 109.45^c^	2786.24 ± 90.77^c^

Averages with the same letter in the same row did not show any significant difference (*P* > 0.05) by Tukey’s test. Values are mean ± standard error. C: 100% beef, TSP15: 15% soy substitution, TSPY15: 15% soy substitution + yeast, TSP30: 30% soy substitution, TSPY30: 30% soy substitution + yeast

### Yield

The yields of the five meatballs are shown in Table 2. There was no significant difference in yield between control and samples with 15% TSP substitution. Samples with 30% TSP substitution show significantly higher yield than control (+ 4.7%) and samples with 15% TSP substitution (+ 3.5–4.9%). There was no significant effect of yeast addition on yield. Deliza et al. (2002) did not report significant differences in the cooking loss of ground beef patties with 15% and 30% TSP substitutions, while Kilic et al. (2010) reported a lower cook loss than control even with a 10% and 20% TSP substitution in beef meatballs. It is possible that such variations might be due to the different recipes, processing and cooking methods used in the studies. The improved cooking yield found with the 30% soy recipes might be related to stronger protein–water interactions created during cooking as well as the increased carbohydrate content.

### Colour

Table 2 shows the colour measurements across the five recipes. There was no significant difference across the five recipes in terms of internal lightness. Control samples had significantly higher internal a* redness values than the other recipes probably because of the higher meat content, while control and TSP15 had significantly lower internal b* yellowness values than the other recipes probably because of the lack of yeast, yellow in colour.

On the exterior, TSPY30 samples had significantly higher lightness values than control and significantly higher b* yellowness values than control and TSP15 samples. The opposite was true for a* redness values, as control had significantly higher redness than the other samples. In general, both on the inside and the outside, a* redness values tended to go down and b* yellowness values tended to go up with soy addition. These results are similar to Deliza et al. (2002), where 15% and 30% TSP substitution resulted in less red beef patties than control. The differences in colour in the present study might be explained by the addition of the TSP and the yeast flakes, as they could both contribute to bringing down the redness of the meatballs and increasing their yellowness. The effect of yeast on colour was significant in the internal b* yellowness between TSP15 and TSPY15 and external b* yellowness between TSP30 and TSPY30, but not between the other colour attributes.

The overall colour difference from control was calculated with ΔE. According to Francis and Clydesdale (1975), if ΔE < 1 the colour differences are not obvious for the human eye, if 1 < ΔE < 3 the colour differences are not appreciative by the human eye, if ΔE > 3 colour differences are obvious for the human eye. The results show that the overall colour difference between control and TSPY30 might be obvious (internal ΔE = 2.9 and external ΔE = 3.8), while all of the other internal and external colour comparisons with control could be considered not obvious or not appreciative by the human eye.

### Texture profile analysis

The addition of soy and yeast had an effect on the texture of meatballs as shown in Table 2. Control and samples with 15% TSP showed similar hardness, while samples with 30% soy were significantly softer than control samples. Similarly, Kassama et al. (2003) found that increased concentration of soy protein significantly decreased hardness in beef patties. In the current study, the addition of increasing levels of soy and yeast might have modified the structure of meatballs resulting in a progressive decrease in hardness and chewiness compared to control. These results may also be linked to the influence of soy on the product moisture (section “Proximate analysis”) and product yield (section “Yield”), and the myofibril-soy protein interaction during the cooking process. It has been reported that the interaction between myofibrillar and soy proteins occurring by heat application, encourages the formation of a gel matrix which has a role in improving the texture in soy-containing meat products (Ramırez-Suárez and Xiong 2003). Some significant springiness and cohesiveness differences across the five recipes were found, but these did not follow a clear pattern.

### Sensory evaluation

#### Liking

Average liking across the five recipe is shown in Table 3. Samples across the five recipes scored above the central scale point (4.5 = neither like nor dislike), although none of the samples received strong ratings. Similar to Kilic et al. (2010), there were no significant differences among the five recipes in terms of appearance liking. Texture results show that control scored significantly lower in acceptability compared to all other samples, which could be related to the high control instrumental hardness seen in section “Texture profile analysis” on TPA. Flavour and overall acceptability both showed TSPY15 scoring significantly higher than control but not significantly higher than the other TSP-containing samples. These results are in contrast with other studies in beef patties, where 20% and 30% TSP inclusions were associated to a significant decrease in overall acceptability compared to control (Kaya and Gökalp 1990; Kilic et al. 2010). Recently Neville et al. (2017) found no significant differences in consumer acceptability between hybrid beef burgers and pork sausages containing up to 37% meat and full-meat commercial samples.

**Table 3:** Mean ratings for appearance, texture, flavour and overall acceptability for the five recipes

Recipe	Appearance	Texture	Flavour	Overall acceptability
C	6.08 ± 0.09^a^	5.12 ± 0.07^b^	5.18 ± 0.07^b^	5.03 ± 0.06^b^
TSP15	5.73 ± 0.08^a^	5.97 ± 0.08^a^	5.78 ± 0.07^ab^	5.68 ± 0.08^ab^
TSPY15	5.73 ± 0.11^a^	6.47 ± 0.10^a^	5.97 ± 0.10^a^	5.95 ± 0.10^a^
TSP30	6.00 ± 0.10^a^	5.90 ± 0.08^a^	5.52 ± 0.07^ab^	5.50 ± 0.08^ab^
TSPY30	5.83 ± 0.09^a^	6.03 ± 0.10^a^	5.48 ± 0.07^ab^	5.48 ± 0.07^ab^

Averages with the same letter in the same column did not show any significant difference (*P* > 0.05) by Tukey’s test. Values are mean ± standard error. C: 100% beef, TSP15: 15% soy substitution, TSPY15: 15% soy substitution + yeast, TSP30: 30% soy substitution, TSPY30: 30% soy substitution + yeast

#### Check-all-that-apply

Cochran’s *Q* test showed significant differences in the frequency with which thirteen out of the twenty-four terms were used to describe the five meatball samples. Then for the thirteen terms that were significantly different, multiple pairwise comparisons were used to understand where difference existed (Table 4).

**Table 4:** Frequency of selection of CATA terms for the five recipes

Attributes	Control	TSP15	TSPY15	TSP30	TSPY30
Appearance					
Moist looking***	35^a^	28^ab^	23^b^	21^bc^	12^c^
Dry looking**	15^b^	20^b^	20^b^	18^b^	32^a^
Uniform colour (outside)^ns^	19	15	20	12	22
Uneven colour (outside)^ns^	18	26	25	24	21
Dark brown (inside)**	17^a^	5^c^	7^bc^	6^bc^	15^ab^
Light brown (inside)***	27^c^	36^ab^	29^bc^	42^a^	25^c^
Unusual^ns^	3	9	5	4	4
Characteristic^ns^	13	8	9	9	15
Texture					
Juicy*	25^ab^	25^ab^	31^a^	20^b^	16^b^
Dry**	23^a^	19^a^	9^b^	20^a^	26^a^
Hard***	25^a^	10^b^	6^b^	5^b^	10^b^
Soft***	7^c^	26^ab^	30^a^	32^a^	19^b^
Solid and difficult to cut***	21^a^	9^b^	7^b^	6^b^	12^ab^
Crumbly and easy to cut***	4^c^	14^b^	18^ab^	25^a^	18^ab^
Unusual^ns^	3	4	4	7	4
Characteristic^ns^	14	11	19	13	16
Flavour					
Tasty^ns^	11	14	23	15	15
Bland**	28^a^	23^a^	14^b^	19^ab^	13^b^
Cheesy*	4^ab^	2^b^	3^ab^	4^ab^	10^a^
Weak meaty^ns^	27	25	25	28	24
Strong meaty^ns^	18	19	20	11	17
Wheat–cereal like***	5^c^	10^c^	11^bc^	22^a^	20^ab^
Unusual^ns^	3	5	6	10	12
Characteristic^ns^	12	11	13	12	11

Cochran’s Q test was used to detect significant differences between terms. ***Indicates significant differences among samples at *P* ≤ 0.001. **Indicates significant differences at *P* ≤ 0.01. *Indicates significant differences at *P* ≤ 0.05. ^ns^Indicates no significant differences (*P* > 0.05). A Sign test was used to make multiple comparisons within each term, with no correction for multiplicity being applied. Different superscript letters (a, b, c) denote significant differences within term (sign test, *P* ≤ 0.05)

In terms of appearance, control samples were the most often associated with the term “moist looking” and the least often associated with the contrasting term “dry looking”, which is in agreement with the moisture results seen in section “Proximate analysis”. The opposite was true for TSPY30 for the terms “moist looking” (least often associated) and “dry looking” (most often associated). A significant difference in colour was also perceived among the five samples. Control and samples containing yeast were the least associated to the term “light brown inside”, while yeast-free samples were the most often associated to “light brown inside”. Control and TSPY30 were more often associated to the opposite term “dark brown inside” than the other samples. This correlates with the instrumental colour differences found in section “Colour” on colour, showing that control samples were significantly redder than the others. No significant differences were detected in the uniformity or unevenness of the meatball external colour across the five recipes, although external ΔE colour differences were detected instrumentally between control and TSPY30. All recipes were also similarly associated to “unusual” and “characteristic” appearance.

Texture was the term category that showed the highest number of differences. TSPY15 was the recipe significantly least associated to “dry” and the recipe associated the most times to “juicy”. Control was the recipe significantly more often associated to “hard” and significantly the least associated to “soft”. These findings support previous work on soy-containing meat products by Deliza et al. (2002) and Liu et al. (1991). Control was also the most selected sample for the term “solid and difficult to cut” and was significantly the least selected sample for the term “crumbly and easy to cut”. It is interesting to note that although control samples were the most associated to “moist looking” and the least associated to “dry looking” based on appearance, upon tasting, this initial perception was not confirmed. All recipes were similarly associated to “unusual” and “characteristic” texture.

In terms of flavour, yeast-containing samples were the least associated to the term “bland” and TSPY30 was the recipe most often associate to “cheesy”. Samples with 30% soy content were more often associated to the term “wheat-cereal like” than the other samples, control was the least associated to this term, while TSP15 and TSPY15 were in the middle, indicating that panellists were able to detect the presence of the different levels of soy in the samples. It is interesting to note that there was no significant difference in the way panellists associated the terms “weak meaty” and “strong meaty” to the five recipes. This is in contrast with Kilic et al. (2010), where a 20% TSP inclusion was associated to a significantly lower perception of meat flavour intensity, but it is in accordance with Neville et al. (2017), where hybrid products were identified as having a “meaty flavour” in line with the meat sausages. In the current study, there was also no significant difference in the way the five recipes were associated to “tasty”, “unusual” and “characteristic” flavour.

#### Correspondence analysis

**Fig. 1:**
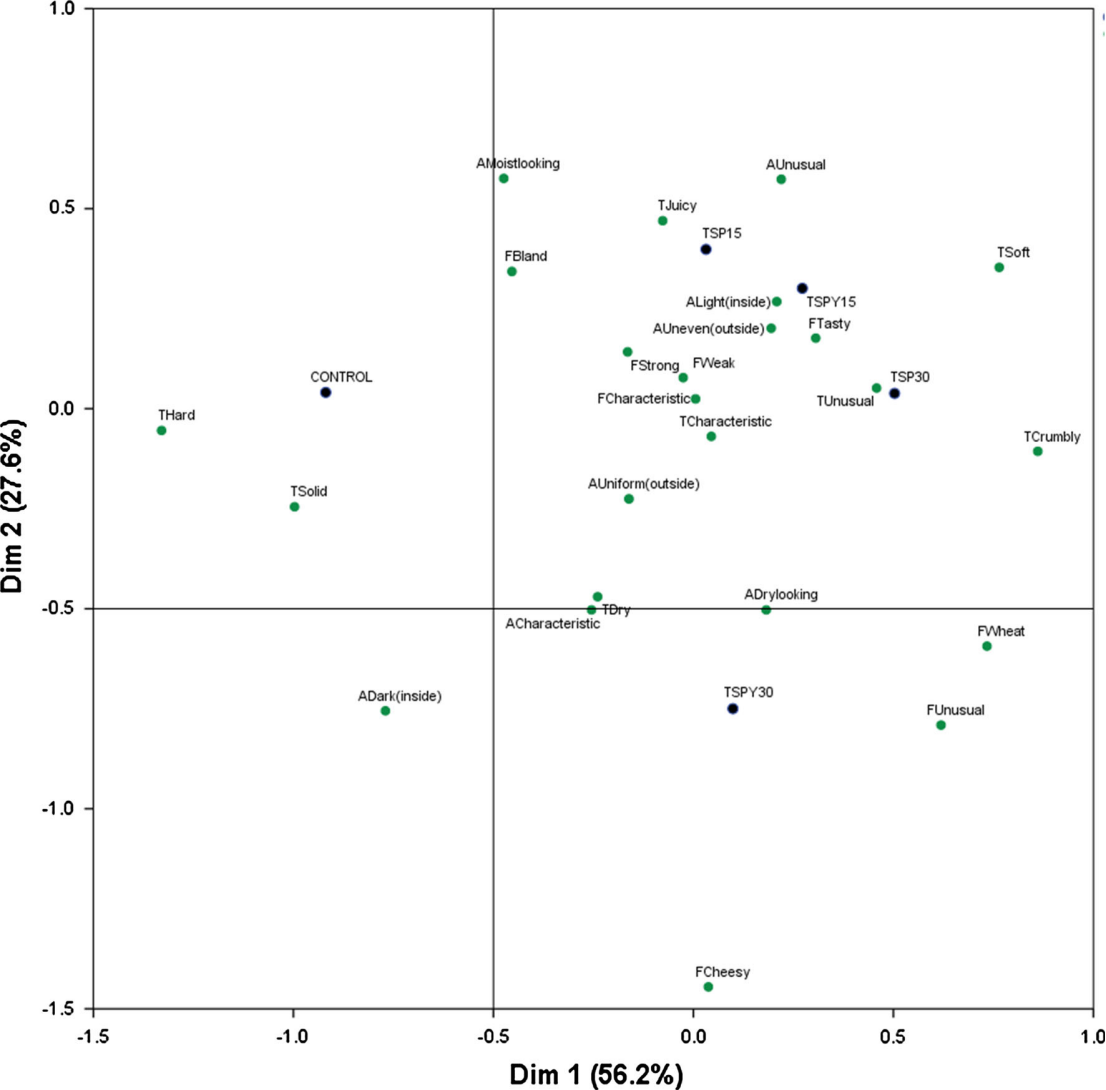
Correspondence analysis (CA) indicating the relationship between the CATA terms used and the five meatball samples. First two dimensions. Attributes relate to appearance (A), flavour (F) and texture (T)

Figure 1 gives a visual representation of the five samples and the twenty-four CATA terms using CA. Combined, the first and second dimensions explained 83.8% of the variance in the data, with a strong first dimension (56.2%) and a weak (less important) second dimension (27.6%). The CA analysis showed that the first dimension was positively correlated with the terms “crumbly and easy to cut”, “soft”, and “wheat-cereal like” and negatively correlated with “hard”, “solid and difficult to cut”. The second dimension was positively correlated with “moist looking” and “unusual” appearance, while it was negatively correlated with “cheesy” flavour.

In CA, the distance between samples is a measure of their similarity (Ares and Jaeger 2015). The sensory map separated the meatballs in three distinct groups: control, located at positive values of the second dimension and negative values of the first dimension; TSPY30, located at negative values of the second dimension and positive values of the first dimension; TSP15, TSPY15 and TSP30 located together at positive values of the first and second dimension, showing similarity. Control was spatially close to “hard” and “solid and difficult to cut”, TSPY30 to “dry” texture, “dry looking” and “unusual” flavour, TSP30 with “unusual” texture, TSPY15 with “tasty” flavour and TSP15 with “juicy” texture.

#### Penalty-lift analysis

**Fig. 2:**
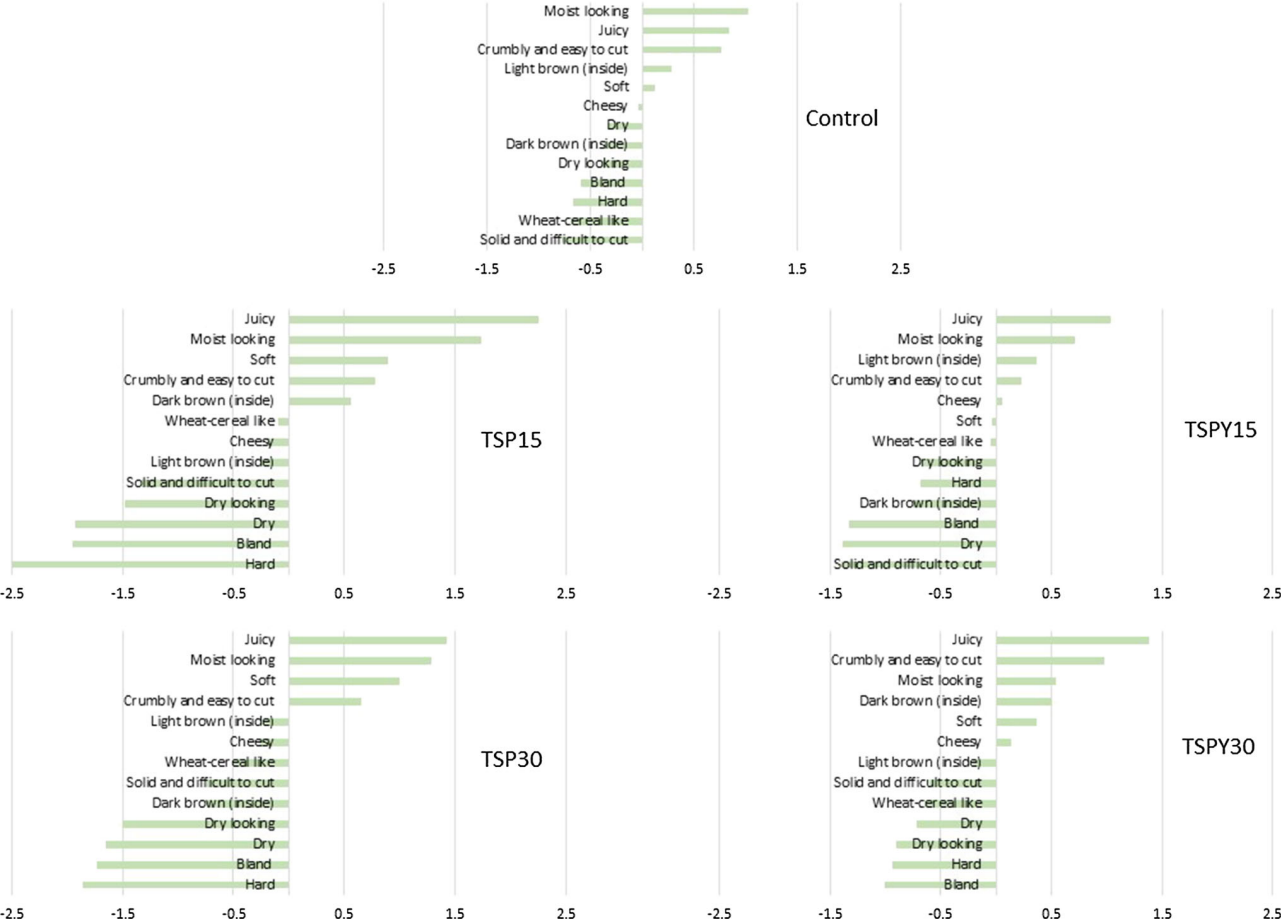
Results of the penalty-lift analysis of the five recipes. The values indicate a change in liking when an attribute was selected compared to when it was not selected by panellists

Penalty-lift analysis was performed to determine which CATA terms had the most impact on liking and to estimate how much liking changed when a term was selected by a panellist compared to when it was not (Meyners and Castura 2014). In order to improve clarity, only terms that significantly discriminated among the five recipes were included in the analysis (Fig. 2).

The main drivers for control liking were “moist looking”, “juicy” and “crumbly and easy to cut”, while the main drivers for dislike were “solid and difficult to cut”, “wheat-cereal like” and “hard”, with liking changing ± 1 point on the liking scale when these were selected. TSP15 and TSP30 had the same three main drivers for like (“juicy”, “moist looking” and “soft”) and the same three main drivers for dislike (“dry”, “bland” and “hard”), but in TSP15 the term impact on liking was more dramatic (up to − 2.5 for “hard” and up to − 2.3 for “juicy”). In TSPY15 “solid and difficult to cut” was the main driver for dislike, while in TSPY30 “crumbly and easy to cut” was the second main driver for dislike. Interestingly, “cheesy” was not a strong driver of like or dislike for any of the samples and “wheat-cereal like” was a driver of dislike only for control (− 0.69) and TSPY30 (− 0.65). Similar to this study, Neville et al. (2017) found that when the terms “juicy”, “easy to cut” and “soft” were not selected in a particular meat hybrid sample, consumer acceptability significantly decreased.

## Conclusion

The present study investigated the effects of replacing 15% or 30% beef by TSP with or without the addition of nutritional yeast on the sensory and instrumental quality of beef meatballs. The addition of TSP and yeast had some effects on the proximate analysis, colour, instrumental texture and sensory profiles. However, increasing texturised soy protein content significantly improved cooking yield and reduced cooking loss. TSPY15 seemed to be the most promising formulation based on the texture, flavour and overall acceptability results. The reduction in meat content did not seem to negatively affect consumer acceptability, with consumer testing showing that the new concept products were generally well accepted by meat eaters compared to control. These results can provide encouragement for the use of the hybrid concept by the meat industry to promote the partial substitution of meat in flexitarians’ diets.
