# Correction to: Effect of texturised soy protein and yeast on the instrumental and sensory quality of hybrid beef meatballs

**DOI:** 10.1007/s13197-019-03763-0

**Published:** 2019-05-07

**Authors:** Simona Grasso, Gabrielle Smith, Sophie Bowers, Oluseyi Moses Ajayi, Mark Swainson

**Affiliations:** National Centre for Food Manufacturing, College of Sciences, University of Lincoln, Lincoln, UK

## Correction to: J Food Sci Technol 10.1007/s13197-018-3552-9

The article “Effect of texturised soy protein and yeast on the instrumental and sensory quality of hybrid beef meatballs”, written by Simona Grasso, Gabrielle Smith, Sophie Bowers, Oluseyi Moses Ajayi, and Mark Swainson, was originally published electronically on the publisher’s internet portal (currently SpringerLink) on 25 February 2018 without open access.

With the author(s)’ decision to opt for Open Choice, the copyright of the article has been changed to © The Author(s) 2019 and the article is forthwith distributed under the terms of the Creative Commons Attribution 4.0 International License (http://creativecommons.org/licenses/by/4.0/), which permits use, duplication, adaptation, distribution and reproduction in any medium or format, as long as you give appropriate credit to the original author(s) and the source, provide a link to the Creative Commons license and indicate if changes were made.

The original article has been corrected.

